# Kinetic effects in thermal explosion with oscillating ambient conditions

**DOI:** 10.1038/s41598-018-22341-6

**Published:** 2018-03-05

**Authors:** Vasily Novozhilov

**Affiliations:** Centre for Environmental Safety and Risk Engineering, Victoria University, Melbourne, VIC 8001 Australia

## Abstract

Thermal explosion problem for a medium with oscillating ambient temperature at its boundaries is a new problem which was introduced in the preceding publication by the present author. It is directly applicable to a range of practical fire autoignition scenarios (e.g. in the storages of organic matter, explosives, propellants, etc.). Effects of kinetic mechanisms, however, need be further investigated as they are expected to alter critical conditions of thermal explosion. We consider several global kinetic mechanisms: first order reaction, second order reaction, and first order autocatalysis. It is demonstrated that kinetic effects related to reactants consumption do indeed shift respective critical boundaries. Effect of kinetics on oscillatory development of thermal explosion is of particular interest. In line with conclusions of the preceding publication, it is confirmed that temperature oscillations may develop during induction phase of thermal explosion when the effect of reactants consumption is properly taken into account. Moreover, development of thermal explosion instability through the prior oscillations is an inevitable and natural scenario. This fact is confirmed by a number of examples. Besides, effects of the other relevant parameter, Zeldovich number on critical conditions are also investigated.

## Introduction

Thermal explosion (thermal runaway or autoignition) is a fundamental combustion science concept describing wide range of observed phenomena. In the preceding publication^1^ an importance of the scenario with ambient temperature variations (oscillations) was identified. Thermal explosion possibility under such conditions must be taken into account when considering safe designs and operations of certain types of facilities, such as, for example, storages of various chemically active solid materials (i.e. biomass or refuse-derived fuels, piles of food grains, coal, wood chips, bagasse and compost; storages containing military munitions, solid propellants, pyrotechnics and similar substances). Fire autoignition risks in such systems are well known and documented^2–8^.

Critical conditions for thermal explosion in the environment with oscillating ambient temperature were quantitatively established in^1^. Moreover, a new scenario of the explosion development was discovered in^1^ where temperature of the media oscillates during induction period, before exhibiting infinite growth. It was also demonstrated that discovered scenarios are practically feasible. Ranges of ambient temperature oscillation frequencies and amplitudes corresponding to practically realizable conditions were established. Daily or yearly ambient temperature variations are highly relevant for the problems involving accidental ignition and subsequent fires of solid materials stored or transported in significant quantities. Therefore, thermal explosion scenarios considered in the publication^1^ as well as in the present paper may well occur in real combustion systems. Development of explosion in practical situations may have quite complicated nature, such as a runaway following a number of temperature oscillations.

It turns out that for the same classes of materials, kinetic effects, such as reactants consumption, or autocatalysis, during the induction stage of the thermal explosion are important. For condensed systems, e.g. propellants, this is stated for example in^9^. It should be noted that studies of influence of kinetic effects on thermal explosion development of any type are rather limited. In most general form this problem was addressed by Babushok *et al*.^10^, Shouman^11^, and Shouman and El-Sayed^12,13^ in classical Semenov formulation without any oscillations in boundary conditions.

The present problem of thermal explosion in oscillatory conditions^1^ is close to the problem of thermal explosion development under linearly increasing ambient temperature^9,14,15^. The relation between the two problems was discussed^1^. Kinetic effects do certainly play a role in the latter problem^9,14,15^. Specifically, at sufficiently low ambient temperature increase rates the reactive medium temperature increases slowly, and eventually kinetic deceleration (due to reactants consumption) compensates chemical reaction self-acceleration, driving a system towards chemical equilibrium. (An exclusion is the reaction of the zero order where reactants consumption is excluded from consideration by definition).

At larger values of the ambient temperature growth rates kinetic factors cannot prevent development of thermal instability. Note that in the basic Semenov and Frank-Kamenetskii thermal explosion problem formulations^16–18^ critical conditions are determined solely by the balance between self-acceleration of the chemical reaction and the rate of heat dissipation. In the case of dynamic regimes with monotonically increasing ambient temperature, mere existence of critical conditions is due to kinetic factors (reactants consumption).

Similarly, as thermal explosion under oscillating ambient conditions represents a profoundly dynamic regime, we expect kinetic factors to play an important role in determining critical conditions for such an explosion. For the purpose of practical applications, kinetic effects must be quantitatively and accurately assessed.

In the present paper, oscillating conditions are coupled with kinetics, which brings new effects into the thermal explosion development picture. In general, since reactants consumption results in slowing the chemical reaction down, inclusion of kinetic effects would lead to the increase of respective critical Frank-Kamenetskii parameters.

For convenience, throughout the paper waved variables refer to dimensional quantities, while unwaved refer to non-dimensional ones.

It is clear from general considerations that the qualitative picture of thermal explosion is not changed by the inclusion of the kinetic effects. Indeed, taking a sufficiently large value $\tilde{\beta } > {\tilde{\beta }}^{\ast }$ (where ${\tilde{\beta }}^{\ast }$ is a critical value^9^) of the ambient temperature linear growth rate would ensure thermal explosion, irrespective of kinetic deceleration. Oscillating conditions are obtained from the latter problem by “bending” the temperature profile at the boundary at later stages (after the thermal explosion have occurred). Therefore, thermal explosion may occur under oscillating ambient temperature conditions in the presence of any particular form of kinetic deceleration. It is equally obvious that if the system happens to be in the state of thermal equilibrium (i.e. in the state below critical for the static ambient conditions) then sufficiently small oscillations cannot lead to thermal instability. Therefore, critical conditions for thermal explosion in the case of oscillating ambient conditions, coupled with kinetic effects can indeed be observed.

Quantitatively, critical conditions in the presently considered formulation may be due to both heat dissipation and kinetic deceleration effects. In the precise form, they should be established by direct computations which are reported later in the present paper.

The aim of the present paper is to extend the study^1^ with the view of providing more realistic assessments of autoignition conditions for practical systems, where kinetic effects are certainly present.

In addition, the effect of possible initial temperature deviation of the reactive media from the ambient temperature is investigated. Such an effect becomes important if due to processing procedures the temperature of the material (for example, biosolid) being deposited into the storage differs from the ambient. Quantitatively, the influence of such an effect on thermal explosion development is being measured by the so-called Zeldovich number defined below.

In the present paper the problem is considered in its most fundamental form in order to achieve major understanding of the process in terms of evaluating effects of the key kinetic parameters. This builds a framework for interpreting behaviours of practical systems, while allowing also the generalization of the results.

On the other hand, the present paper provides important theoretical findings, in particular related to occurrence of oscillations during induction period of thermal explosion, in the presence of kinetic effects.

## Mathematical model

In the classical thermal explosion formulation, due to Frank-Kamenetskii^9,17,18^, the following set of governing equations is considered

1 $$\tilde{\rho }{\tilde{c}}_{p}\frac{\partial \tilde{T}}{\partial \tilde{t}}=\tilde{\lambda }\frac{{\partial }^{2}\tilde{T}}{\partial {\tilde{x}}^{2}}+\tilde{Q}\tilde{\dot{w}}$$

2 $$\frac{d\tilde{C}}{d\tilde{t}}=-\tilde{\dot{w}}$$

The first of these equations describe conservation of energy in the system by means of the heat transfer equation with the source term. Here $\tilde{T}$, $\tilde{t}$, $\tilde{x}$, $\tilde{\rho }$, ${\tilde{c}}_{p}$, $\tilde{\lambda }$ stand for temperature, time, cartesian coordinate, density, specific heat and thermal conductivity, respectively. The source term describes the rate of energy generation in the system and is a product of the heat of reaction $\tilde{Q}$ and the reaction rate

3 $$\tilde{\dot{w}}={\tilde{C}}^{n}{\tilde{k}}_{0}(\tilde{T})$$

The function ${\tilde{k}}_{0}(\tilde{T})$ in the equation (3) is called a reaction rate constant and depends on temperature in the form of specific Arrhenius function

4 $${\tilde{k}}_{0}(\tilde{T})=\tilde{A}\exp (-\frac{\tilde{E}}{\tilde{R}\tilde{T}})$$

where $\tilde{A}$ is pre-exponential factor, $\tilde{E}$ activation energy, $\tilde{R}$ universal gas constant.

Below we also make use of the modified rate constant defined here as $\tilde{k}(\tilde{T})={\tilde{C}}_{0}^{n}{\tilde{k}}_{0}(\tilde{T})$.

The equation (2) is the mass conservation (kinetic) equation which describes the fact that the reactant with the concentration $\tilde{C}$ is consumed in chemical reaction at the certain reaction rate.

For the purpose of further analysis the problem is formulated in non-dimensional form, making use of the following scales:

$\tilde{R}{\tilde{T}}_{0}^{2}/\tilde{E}$(characteristic temperature interval of the chemical reaction) for temperature deviation from the initial medium temperature, $\tilde{L}$ (characteristic size of reactive material) for spatial scale; ${\tilde{t}}_{ad}=\frac{\tilde{\rho }{\tilde{c}}_{p}\tilde{R}{\tilde{T}}_{0}^{2}}{\tilde{Q}\tilde{E}\tilde{k}({\tilde{T}}_{0})}$ (adiabatic time scale) for time. Mean value of the oscillating ambient temperature is indicated by the subscript “0”, and the modified reaction rate constant at this mean value is denoted by $\tilde{k}({\tilde{T}}_{0})$.

Using the above scaling, the following two non-dimensional equations, namely the heat transfer equation and the kinetic equation are obtained:

5 $$\frac{{\rm{\partial }}\theta }{{\rm{\partial }}\tau }=\frac{1}{\delta }(\frac{{{\rm{\partial }}}^{2}\theta }{{\rm{\partial }}{\xi }^{2}}+\frac{m}{\xi }\cdot \frac{{\rm{\partial }}\theta }{{\rm{\partial }}\xi })+\exp (\frac{\theta }{1+Ar\cdot \theta })\cdot \phi \,(\eta )\,\,\,\,\,\,\,0\le \xi \le 1;\,\tau \ge 0$$

6 $$\frac{d\eta }{d\tau }=Td\cdot \exp (\frac{\theta }{1+Ar\cdot \theta })\cdot \phi (\eta )\,\,\,\,\,\,\,\tau \ge 0$$

where *θ* is temperature, *τ* is time, *ξ* is spatial coordinate, $\delta =\frac{\tilde{Q}\tilde{\rho }\tilde{k}({\tilde{T}}_{0})\tilde{E}{\tilde{L}}^{2}}{\tilde{\lambda }\tilde{R}{\tilde{T}}_{0}^{2}}$ is Frank-Kamenetskii parameter, $Ar=\frac{\tilde{R}{\tilde{T}}_{0}}{\tilde{E}}$ is Arrhenius number, and $Td=\frac{\tilde{\rho }{\tilde{c}}_{p}\tilde{R}{\tilde{T}}_{0}^{2}}{\tilde{Q}\tilde{E}{\tilde{C}}_{0}}$ is Todes number (the ratio of the adiabatic time scale, introduced earlier, and the isothermal time scale $\frac{{\tilde{C}}_{0}}{\tilde{k}({\tilde{T}}_{0})}$).

Kinetic equation is written for the progress variable $\eta =1-\tilde{C}/{\tilde{C}}_{0}$ where ${\tilde{C}}_{0}$ is initial reactant concentration. The kinetic function *φ*(*η*) that appears in the equations (5, 6) may take different specific forms. In the present paper we consider the following kinetic models:

7 $$\phi (\eta )={(1-\eta )}^{n}$$

reaction of the n-th order, and we are considering either *n* = 1 (first order) or *n* = 2 (second order) in the present study

8 $$(\eta +{\eta }_{0})(1-\eta )$$

first order autocatalysis

Kinetic function is a monotonically decreasing function of the progress variable, except for the autocatalytic reaction where kinetic function have a maximum at some *η* > 0.

Various symmetrical cases, with the symmetry index *m* = 0 (planar symmetry), *m* = 1 (cylindrical symmetry) or *m* = 2 (spherical symmetry) may be considered for the set of equations (5, 6).

Due to space limitation, only results for the planar symmetry case *m* = 0 are presented. This case describes a slab of material between the two parallel planes where the temperature only varies in the direction normal to the planes.

The two types of physically relevant boundary conditions may be written in the following form

9 $$\theta (1,\tau )=Zn+{A}_{0}\,\sin (\omega \tau )$$

(the first kind) and

10 $$\frac{\partial \theta }{\partial \xi }(1,\tau )=Zn+Bi\,(\theta -{A}_{0}\,\sin (\omega \tau ))$$

(the second kind, i.e. Newtonian heat exchange)

where $A{}_{0}=\frac{{\tilde{A}}_{0}}{(\tilde{R}{\tilde{T}}_{0}^{2}/\tilde{E})}$ and $\omega ={\tilde{t}}_{ad}\cdot \tilde{\omega }$ are non-dimensional amplitude and frequency of the temperature oscillations, corresponding to the respective dimensional variables ${\tilde{A}}_{0}$ and $\tilde{\omega }$, *Bi* is Biot number.

Zeldovich number $Zn=\frac{{\tilde{T}}_{a}-{\tilde{T}}_{0}}{\tilde{R}{\tilde{T}}_{0}^{2}/\tilde{E}}$ measures the ratio of the difference between ambient and initial material temperatures to the characteristic temperature interval of the chemical reaction.

The symmetry is enforced by the following condition

11 $$\frac{\partial \theta }{\partial \xi }(0,\tau )=0$$

Behaviour of the system is controlled by the following set of parameters. The most important is the Frank-Kamenetskii parameter *δ*, describing critical conditions for the system evolution. This parameter is being assigned a subcritical (for the static *A*_0_ = 0 conditions) value. The positive difference between its critical value *δ*_*cr*_ at static ambient conditions and the actual value describes the degree of subcriticality of the system.

Other parameters are Arrhenius number *Ar*, Todes number *Td*, Zeldovich number *Zn*, oscillation amplitude and frequency *A*_0_, *ω* and, in case of boundary conditions of the second kind, also Biot number *Bi*.

Therefore, we have the following sets of controlling parameters:

(*δ*, *Ar*, *Td*, *Zn*, *A*_0_, *ω*) - for the boundary conditions of the first kind;

(*δ*, *Bi*, *Ar*, *Td*, *Zn*, *A*_0_, *ω*) - for the boundary conditions of the second kind.

Critical conditions are described in terms of dependences *A*_0_ (*ω*) at the critical boundary with all the other parameters being fixed. Since the parameter *δ* is assigned a subcritical value, then the procedure is to increase the amplitude *A*_0_ (at fixed frequency *ω*) until some critical value of the amplitude is reached. Such a procedure correctly determines position of the critical boundary.

Equation (5) is solved numerically using Crank-Nicolson scheme with a central difference approximation for the second derivative (only *m* = 0 is considered). Equation (6) is solved using implicit, first order in time difference scheme. Within each time step, an appropriate number of sub-iterations are performed to achieve convergence to a coupled solution satisfying both (5) and (6). To ensure appropriate level of grid-independence, spatial and time steps are refined to the point where relative error in output values does not exceed 10^−6^. Typically, ten internal sub-iterations are required within each time step.

Critical boundary *A*_0_ (*ω*) is determined by separating infinitely growing solutions from the bounded solutions. In the present intrinsically non-linear problem this can only be done numerically, to a certain accuracy.

The procedure followed in the present study is similar to^1^. Infinitely growing, corresponding to explosion conditions, temperature history profiles have an inflection point. First, the time interval [0, τ^*^] is fixed and the amplitude is being increased until an inflection point appears at *τ* = τ^*^. Such an amplitude provides an estimate for the exact position *A*_0_ (*ω*) of the critical boundary. To limit the dependence of this estimate on the actual value of τ^*^, similar estimation is obtained using the interval [0, 2τ^*^]. The parameter τ^*^ is increased until the relative error between the two estimates falls within 2%. The amplitude corresponding to the [0, 2τ^*^] estimate is taken as a final position of the critical boundary. A typical value of τ^*^ required to obtain the data presented in the Results and discussion section below is around 200.

## Results and Discussion

The problem under consideration is apparently multi-parametric, with the critical conditions being determined by six parameters in the case of the boundary conditions of the first kind, and by seven parameters for the boundary conditions of the second kind.

In the view of these circumstances, most important dependences which supply the essential information about the system behaviour, both qualitatively and quantitatively, need be chosen for reporting in the paper of a limited volume. To this extent, the most important parameters that affect position of a critical boundary and are being varied in the present study are reported in the Table 1 below.

**Table 1:** Case matrix for parametric analysis of critical conditions.

Parameter	Range of variation	Boundary conditions	Reporting item
Oscillation frequency *ω*	0.00–10.00	First kind Second kind	Figs 1–3
Biot number *Bi*	5.00–20.00	Second kind	Figs 2 and 3
Arrhenius number *Ar*	0.00–0.30	Second kind	Fig. 4
Todes number *Td*	0.00–0.20	First kind Second kind	Figs 5 and 6
Zeldovich number *Zn*	−2.00–2.00	Second kind	Fig. 7

In choosing the variation range for each of the parameters primary consideration was to ensure that the entire practically applicable range is fully covered. In some cases, this implies substantial widening of the parameters ranges in order to accommodate not only classical combustion systems but also modern fuels.

In interpreting the figures below it is reminded again that, in accordance with the notation convention in the present paper, the both variables *A*_0_ and *ω* (unwaved!) are non-dimensional.

**Figure 1:**
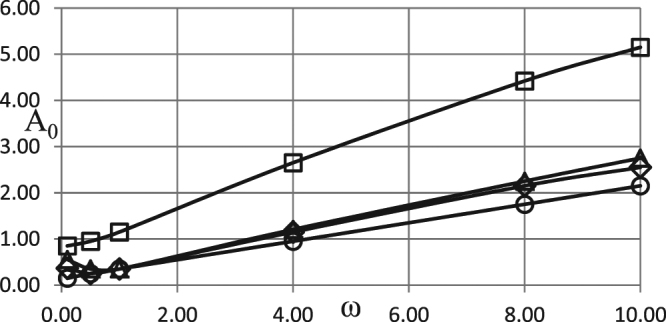
Critical dependences *A*_0_ (*ω*). Boundary conditions (BC) of the first kind. *δ* = 0.8; *Ar* = 0.025; *Td* = 0.005; *Zn* = 0.0; *η*_0_ = 0.5 Ο - zero order (no kinetic effects); ◊ - first order kinetics; Δ - second order kinetics; □ - first order autocatalysis.

In making analysis of the kinetic effects we start with the consideration of the boundary conditions of the first kind. There are four controlling parameters, apart from the amplitude and frequency, in this case. These are Frank-Kamenetskii parameter as well as the Arrhenius, Todes and Zeldovich numbers. Dependences of critical conditions on different kinetic schemes are compared in Fig. 1. The Frank-Kamenetskii parameter is fixed at *δ* = 0.8, below the critical one for the constant unperturbed ambient temperature case *A*_0_ = 0, which is as well known^18^ *δ*_*cr*_ = 0.88. The values of all the other fixed parameters are provided in the Fig. 1 caption.

As evident from Fig. 1, the dependences given by the first order and the second order kinetics are very close to the case of zero order kinetics. This suggests that the effect of reactants consumption (close to critical conditions) is rather small.

In contrast, autocatalysis conditions exert the major effect on the critical conditions for thermal explosion. Respective values of the critical Frank-Kamenetskii parameter are significantly higher than in case with no kinetics. This is due to the fact that the reaction in this case is slow initially (at small values of the progress variable), and then grows (until the maximum is reached at *η* = (1 − *η*_0_)/2) due to accumulation of the products of the reaction.

As expected, the curve corresponding to the first order kinetics lies above the one corresponding to no kinetics case, and in turn below the curve corresponding to the second order kinetics (increased degree of reactant consumption makes explosion more difficult to occur). The least favourable conditions for thermal explosion correspond to the autocatalytic reaction where deceleration of chemical transformation is most significant.

It is also evident from Fig. 1 that, except for the range of low frequencies 0 < *ω* ≤ ≈ 1 for the zero-, first- or second- order kinetics critical dependences of the amplitude on the frequency are very close to linear. Low frequencies case is however the most important for practical applications^1^.

An important conclusion following from Fig. 1 is that, in line with the results of the study^1^, the system that is thermally stable under constant ambient conditions, may develop instabilities (thermal explosion) if ambient conditions vary in oscillating manner, even if chemical reaction is being damped by reactants consumption.

**Figure 2:**
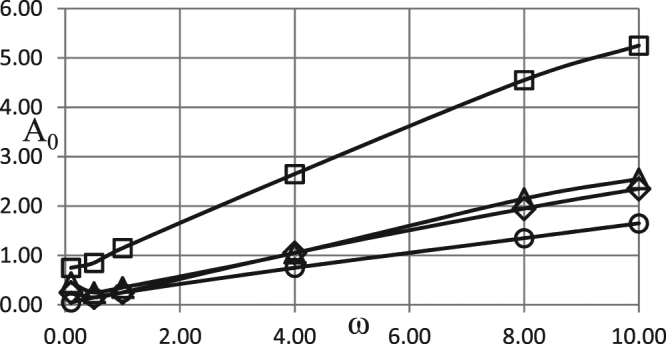
Critical dependences *A*_0_ (*ω*). BC of the second kind. *δ* = 0.6; *Ar* = 0.025; *Td* = 0.005; *Zn* = 0.0; *Bi* = 5.0; *η*_0_ = 0.5 Ο - zero order kinetics; ◊ - first order kinetics; Δ - second order kinetics; □ - first order autocatalysis.

**Figure 3:**
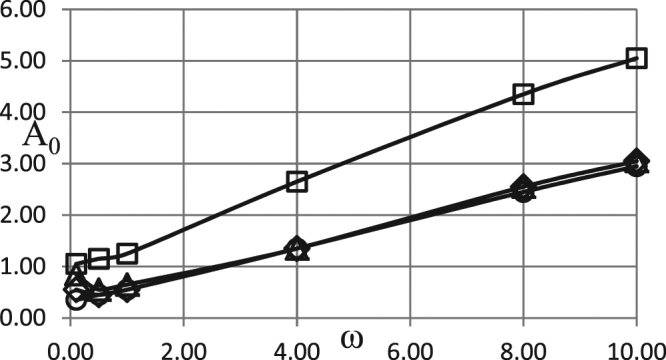
Critical dependences *A*_0_ (*ω*). BC of the second kind. *δ* = 0.6; *Ar* = 0.025; *Td* = 0.005; *Zn* = 0.0; *Bi* = 20.0; *η*_0_ = 0.5. Ο - zero order kinetics; ◊ - first order kinetics; Δ - second order kinetics; □ - first order autocatalysis.

Similar trends are observed in the simulations with the boundary conditions of the second kind. The corresponding results are presented in Figs 2 and 3 for the two different intensities of heat losses, i.e. for different values of the parameter *Bi*.

The critical curve for autocatalytic reaction is positioned significantly higher than the other curves, indicating much higher critical Frank-Kamenetskii parameters for thermal explosion. This is due to the fact that significant pre-accumulation of the reaction products is needed before reaction acquires considerable rate required for explosion to occur. Such pre-accumulation requires significant ambient temperature increase (i.e. significant amplitude of oscillations) during induction period in order to heat the material up and provide noticeable reaction rate generating reaction products.

Higher intensity of heat losses for the case presented in Fig. 3 results in higher critical Frank-Kamenetskii parameters, compared to Fig. 2, for the zero-, first- or second- order kinetic mechanisms. The critical boundary for the autocatalytic reaction is not affected by the change in *Bi* numbers. Again, this is due to fundamental difference of the autocatalytic mechanism from the other mechanisms, where in the former case heat generation (controlled by reaction rate) is slow during the induction period, and therefore not significantly affected by changing heat loss conditions at the boundary.

Simulation results presented in Figs 1–3 demonstrate essentially linear dependences of the critical ambient temperature oscillation amplitudes on the frequency of oscillations. Explanation for such behaviour is as follows. Thermal stability of the system is effectively determined by its behaviour at the temperatures close to the maximum (through oscillation cycle). If this maximum temperature is fixed, then thermal explosion induction period (at this temperature) becomes larger than the period of oscillations as the frequency of the latter increases. Consequently, along the critical boundary, oscillation amplitude must increase with the increase of frequency. Constant rate of increase indicates that along the critical boundary product of the maximum oscillation temperature and the time over which ambient temperature stays close to this maximum remains nearly constant. The latter time is inversely proportional to the frequency of oscillations.

It is instructive to interpret critical conditions identified in the Figs 1–3 in terms of dimensional parameters. Recalling the definitions of non-dimensional amplitude and frequency, the conditions representing risk of thermal explosion may be expressed in the space of real physical variables as ${\tilde{A}}_{0}\,\, > \,\,(\tilde{R}{\tilde{T}}_{0}^{2}/\tilde{E})f(\frac{\tilde{\rho }{\tilde{c}}_{p}\tilde{R}{\tilde{T}}_{0}^{2}}{\tilde{Q}\tilde{E}\tilde{k}({\tilde{T}}_{0})}\tilde{\omega })$ where *f*(*ω*) represents any relevant critical curve from the Figs 1–3. The above inequality estimates the risk for a system with particular thermophysical and kinetic properties experiencing ambient temperature oscillations with given amplitude and frequency. It should be noted that the realistic values for the latter parameters are *A*_0_ ≤ ≈ 20 °*C* (overall temperature variation within $\approx 40{}^{\circ }C$), and $6\cdot {10}^{-7}{s}^{-1}\le \approx \omega \le \approx 6\cdot {10}^{-5}{s}^{-1}$ (daily or seasonal temperature variations)^1^.

**Figure 4:**
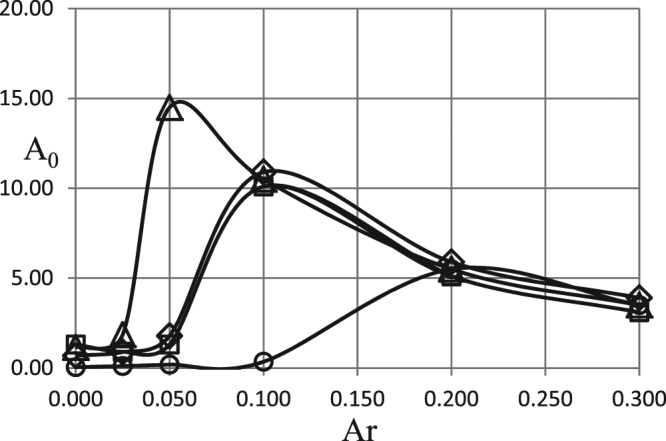
Critical dependences *A*_0_ (*Ar*). BC of the second kind. *ω* = 0.5 *δ* = 0.6; *Td* = 0.05; *Zn* = 0.0; *Bi* = 5.0; *η*_0_ = 0.5. Ο - zero order kinetics; ◊ - first order kinetics; Δ - second order kinetics; □ - first order autocatalysis.

Effect of the Arrhenius number is illustrated in Fig. 4. For the classical combustion systems (say gaseous flame) Arrhenius number is a small parameter (normally <0.1). The fuels considered in the present study, however, may exhibit smaller activation energy. Therefore, it is relevant to consider extended range of Arrhenius numbers. This is done in Fig. 4. The value of the oscillation frequency is fixed in the most practically relevant range^1^.

Dependences of the critical amplitude on the Arrhenius number reveal quite peculiar behaviour. As evident from the set of equations (5, 6) the two competitive effects appear when varying Arrhenius number. The rate of chemical reaction decreases with increase of the Arrhenius number. Therefore, a demand of the energy generation sufficient for thermal runaway would dictate larger critical oscillation amplitudes. On the other hand, consumption of reactants drops along with the decrease of the reaction rate. This effect contributes to the decrease of the critical amplitude with the rising Arrhenius number.

The competition between the two effects results in the curves having an extremum (maximum) in Fig. 4. In the range of small Arrhenius numbers the dependence of the heat generation rate on this parameter is more important; the critical amplitude increases with increase of the Arrhenius number. This is consistent with the preceding results^1^ where the Fig. 2 demonstrates the same trend. As the Arrhenius number increases further the kinetic effect (decrease in the reactants consumption rate) becomes dominant. The critical amplitude gradually decreases. As the effect of the reactants consumption decreases it must be expected that the dependence of the critical amplitude on the Arrhenius number eventually becomes independent of the particular kinetic mechanism. This is exactly what is seen in Fig. 4, where the curves corresponding to different kinetic mechanisms collapse onto the curve corresponding to the absence of any kinetic effects (zero order reaction) for $Ar > \approx 0.2$, thus confirming consistency of physics captured by the model.

It is instructive to consider also the effects of the other controlling parameters, namely Todes and Zeldovich numbers on the shapes and positions of critical boundaries.

**Figure 5:**
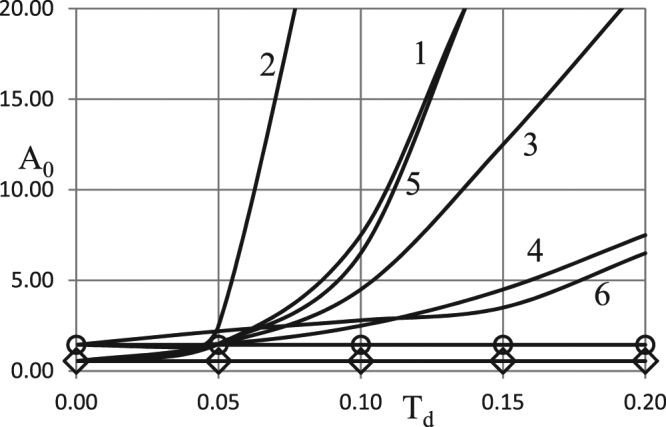
Critical dependences *A*_0_ (*Td*). BC of the first kind. *δ* = 0.6; *Ar* = 0.025; *Zn* = 0.0; *Bi* = 5.0; *η*_0_ = 0.5 ◊ - zero order kinetics, *ω* = 0.5; Ο - zero order kinetics, *ω* = 4.0. 1 - first order kinetics, *ω* = 0.5; 2 - second order kinetics, *ω* = 0.5; 3 - first order autocatalysis, *ω* = 0.5; 4 - first order kinetics, *ω* = 4.0; 5 - second order kinetics, *ω* = 4.0; 6 - first order autocatalysis, *ω* = 4.0.

**Figure 6:**
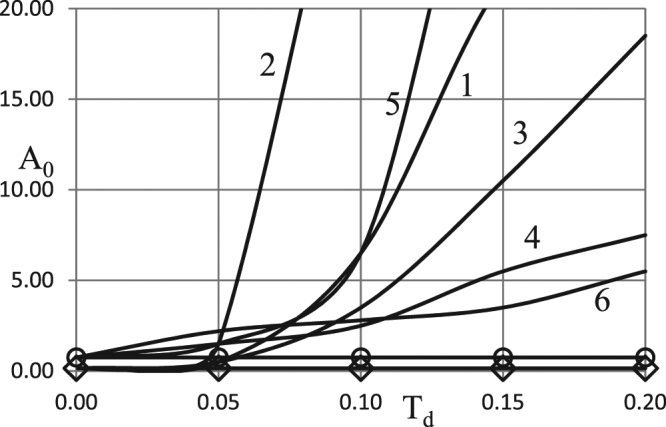
Critical dependences *A*_0_ (*Td*). BC of the second kind. *δ* = 0.6; *Ar* = 0.025; *Zn* = 0.0; *Bi* = 5.0; *η*_0_ = 0.5 ◊ - zero order kinetics, *ω* = 0.5; Ο - zero order kinetics, *ω* = 4.0. 1 - first order kinetics, *ω* = 0.5; 2 - second order kinetics, *ω* = 0.5; 3 - first order autocatalysis, *ω* = 0.5; 4 - first order kinetics, *ω* = 4.0; 5 - second order kinetics, *ω* = 4.0; 6 - first order autocatalysis, *ω* = 4.0.

First we examine effects of the Todes number variation. The results of the analysis are presented in Figs 5 and 6 for the two types of boundary conditions and the two different values of the oscillation frequency.

Again, as in the case with the Arrhenius number variation, it is necessary to consider an extended range for this parameter, compared to the classical combustion systems ($Td < \approx 0.015$).

Trends observed in Figs 5 and 6 are compatible with the qualitative argument that increase in the Todes number leads to higher rate of reactants consumption (equation (6)). Consequently, critical oscillation amplitude (at fixed oscillation frequency) must increase as the Todes number increases.

The rate of such increase is generally higher at low frequencies (slow ambient temperature variation), such as $\omega =0.5$ in Figs 5 and 6, as at low frequencies the system spends more time near the maximum of the ambient temperature curve.

Another conclusion drawn from comparison between Figs 5 and 6 is that the system exhibits little sensitivity to the type of the boundary conditions imposed at the outer boundary of the system (first or second kind). This is consistent with the earlier results^1^ (e.g. relatively small difference between corresponding curves for different types of the boundary conditions in Figs 2 and 5^1^; weak dependence on the Biot number, Fig. 4^1^).

**Figure 7:**
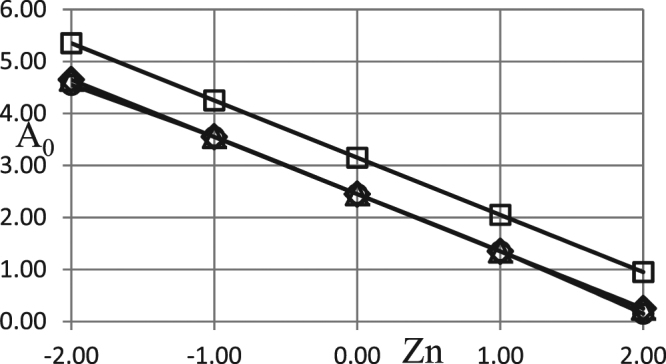
Critical dependences *A*_0_ (*Zn*). BC of the second kind. *ω* = 4.0 *δ* = 0.1; *Ar* = 0.025; *Td* = 0.005; *Bi* = 5.0; *η*_0_ = 0.5. Ο - zero order kinetics; ◊ - first order kinetics; Δ - second order kinetics; □ - first order autocatalysis.

Influence of the Zeldovich number $Zn=\frac{{\tilde{T}}_{a}-{\tilde{T}}_{0}}{\tilde{R}{\tilde{T}}_{0}^{2}/\tilde{E}}$ is illustrated in Fig. 7.

Practically important range of Zeldovich numbers may be estimated in the following way. Taking the typical value of the Arrhenius number as $Ar=\tilde{R}{\tilde{T}}_{0}/\tilde{E}=0.03$, it can be easily seen that the characteristic temperature interval (at the ambient temperature of 300 *K*) would be of the order of $\tilde{R}{\tilde{T}}_{0}^{2}/\tilde{E}\approx 10\,K$. Entering, according to the definition of the Zeldovich number, this characteristic temperature interval $\tilde{R}{\tilde{T}}_{0}^{2}/\tilde{E}$ into the denominator leads to straightforward conclusion that the change in Zeldovich number value of the order of unity corresponds to approximately 10 °C difference between the initial ambient temperature and initial temperature of reacting material. Therefore, the range of the Zeldovich number variation in Fig. 7 covers the range of ambient temperature variations within approximately 40 °C. This is a realistic upper estimation for seasonal temperature variations. Daily variations are typically much smaller. Further, choosing higher value of the Arrhenius number, used to make the estimation, would lead to more narrow range of Zeldovich numbers corresponding to the above upper variation limit (i.e. ≈40 °C). Therefore, $-\,2\le Zn\le 2$ represents the widest practically relevant range for Zeldovich numbers.

Positive values of Zeldovich number indicate the cases of additional heat supply from surroundings to the system, leading to substantial decrease of the critical Frank-Kamenetskii parameters. In the opposite case of negative Zeldovich numbers the system initially loses heat into the lower temperature surroundings; the amplitude of temperature oscillations needed to drive the system towards thermal explosion increases, hence critical Frank-Kamenetskii parameters become higher.

The actual shape of dependences in Fig. 7 is perfectly linear indicating that heat flux transferred between the system and its surroundings (and proportional to *Zn*) plays decisive role in determining critical conditions. Indeed, influence of ambient temperature oscillations is being felt by the system over finite time scale, while the effect of the heat flux controlled by Zeldovich number is being exerted on the system instantaneously.

Of particular significance is discovered in^1^ oscillatory mechanism of transition to explosion where temperature oscillations within the media occur during the induction period of thermal explosion. Here we quantify implications of different kinetic mechanisms on such a scenario. It turns out that this type of thermal instability development is not only possible for any of the kinetic schemes considered but represents an inevitable scenario upon gradual smooth transition of controlling parameters across the stability boundary.

**Figure 8:**
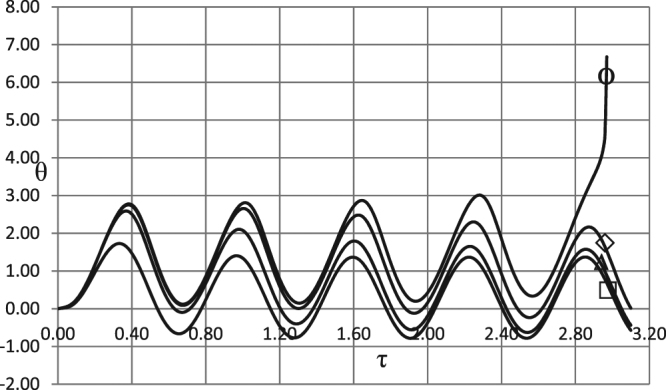
Degeneration of thermal explosion under kinetic conditions. Temperature time history at *ξ* = 0. BC of the first kind. *δ* = 0.6; *Ar* = 0; *Td* = 0.005; *Zn* = 0.0; *η*_0_ = 0.5; *ω* = 10.0; *A*_0_ = 2.707. Ο - zero order kinetics; ◊ - first order kinetics; Δ - second order kinetics; □ - first order autocatalysis.

We consider first kinetic effects in the case similar to the one presented in Fig. 8 of the publication^1^. Such the case is shown in Fig. 8 below by the curve corresponding to the absence of kinetic effects.

In this case reactants consumption clearly transform the system from supercritical to subcritical conditions. Magnitude of instability damping effect increases from first order to second order kinetics, and further to autocatalysis kinetics.

**Figure 9:**
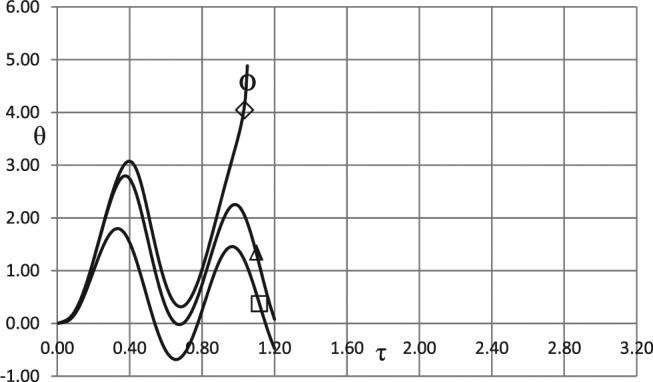
Thermal explosion development under different kinetic mechanisms. Temperature time history at *ξ* = 0. BC of the first kind. *δ* = 0.6; *Ar* = 0; *Td* = 0.005; *Zn* = 0.0; *η*_0_ = 0.5; *ω* = 10.0; *A*_0_ = 2.80. Ο - zero order kinetics; ◊ - first order kinetics; Δ - second order kinetics; □ - first order autocatalysis.

**Figure 10:**
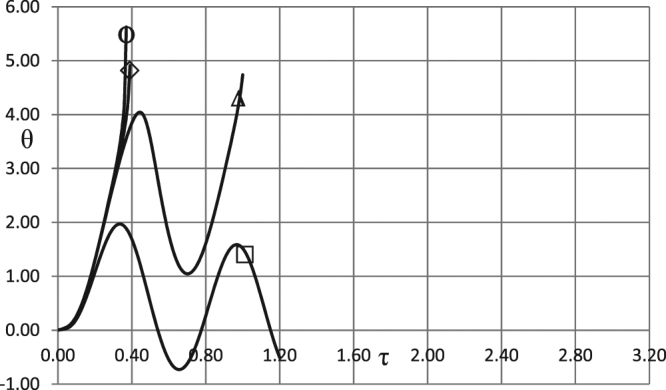
Thermal explosion development under different kinetic mechanisms. Temperature time history at *ξ* = 0. BC of the first kind. *δ* = 0.6; *Ar* = 0; *Td* = 0.005; *Zn* = 0.0; *η*_0_ = 0.5; *ω* = 10.0; *A*_0_ = 3.02. Ο - zero order kinetics; ◊ - first order kinetics; Δ - second order kinetics; □ - first order autocatalysis.

**Figure 11:**
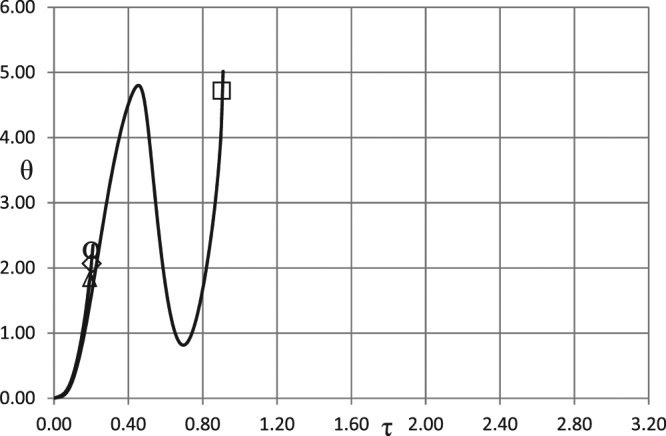
Thermal explosion development under different kinetic mechanisms. Temperature time history at *ξ* = 0. BC of the first kind. *δ* = 0.6; *Ar* = 0; *Td* = 0.005; *Zn* = 0.0; *η*_0_ = 0.5; *ω* = 10.0; *A*_0_ = 4.32. Ο - zero order kinetics; ◊ - first order kinetics; Δ - second order kinetics; □ - first order autocatalysis.

Such damping scenario is, in fact, not the only possibility. Consider what happens if controlling parameters are varied in such a way that critical boundary is crossed. Once the boundary is crossed, the instability may develop in two possible ways: temperature grows infinitely either in monotonic fashion, or through a number of oscillations. The series of Figs 9–11 demonstrates that both mechanisms of stability loss occur.

Figure 9 shows oscillatory explosion development for the zero order (absence of kinetic effects) and the first order mechanisms. By comparing this result to Fig. 8, where the only case leading to instability is the zero-order mechanism, one can see that increasing the value of the amplitude from *A*_0_ = 2.707 to *A*_0_ = 2.80 causes yet another mechanism (first order kinetics) to develop instability of oscillatory type.

Increasing the amplitude further we see first (Fig. 10) that zero- and first order kinetics start to exhibit monotonic development to instability, while second order mechanisms changes behaviour from stable to oscillatory unstable.

Upon further increase of the value of the amplitude behaviour of the autocatalytic mechanism changes to monotonic as well.

Interpreting the pattern apparent from Figs 9–11, we arrive at the conclusion that for any kinetic mechanism the loss of stability, upon entering the unstable region, occurs first in oscillatory manner, following (as controlling parameters move deeper into the region of instability) by monotonic instability development.

Such a picture has rather universal nature as it can be observed in other combustion systems, for example in a loss of stability of solid propellant combustion regimes^19^. In this case, propellant burning rate, upon crossing the boundary of the stable combustion regime, experience oscillations with infinitely growing amplitude, and further into the instability region grows infinitely in monotonic fashion.

**Figure 12:**
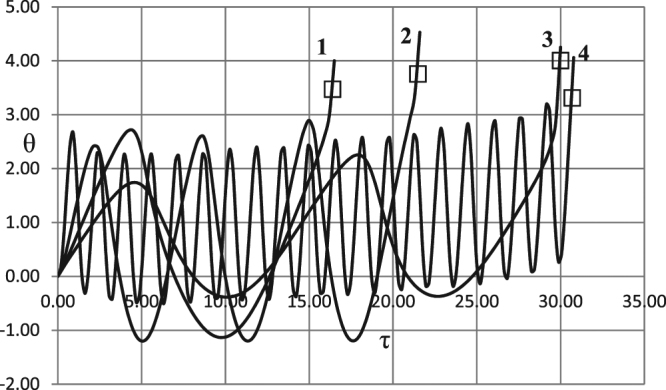
Oscillatory explosion development for autocatalytic reaction. Temperature time history at *ξ* = 0. □ - first order autocatalysis. BC of the second kind. *Ar* = 0.025; *Td* = 0.005; *Zn* = 0.0. 1 - *δ* = 0.6; *Bi* = 20.0; *ω* = 0.5; *A*_0_ = 1.2. 2 − *δ* = 0.6; *Bi* = 20.0; *ω* = 1.0; *A*_0_ = 1.3. 3 − *δ* = 0.8; *Bi* = 5.0; *ω* = 0.5; *A*_0_ = 0.6. 4 − *δ* = 0.8; *Bi* = 5.0; *ω* = 4.0; *A*_0_ = 2.4.

The fact that oscillatory transition to explosion is not an exceptional but rather common scenario is also evidenced by various cases, presented in Fig. 12 for autocatalytic reaction, for various frequencies and amplitudes. Autocatalytic kinetic mechanism exhibits especially wide spectrum of oscillatory scenarios due to the fact that kinetic function in this case has critical (maximum) point and therefore possesses some kind of intrinsic “frequency”. This intrinsic frequency may resonate with frequencies of externally imposed ambient temperature oscillations, giving rise to versatile oscillatory scenarios of transition to explosion.

Further, in Fig. 11 (*A*_0_ = 4.32) the change in the second order mechanism behaviour is observed as it transforms from oscillatory instability (Fig. 10) to monotonic instability development.

The present study provides comprehensive analysis of thermal explosion development under oscillatory ambient conditions, properly taking into account the effects of reactants consumption. The results presented above demonstrate that for any type of kinetic mechanism reacting materials may develop thermal explosion instabilities if ambient conditions (temperature) vary in oscillating manner with appropriate frequency and amplitude. In other words, consumption of reactants (kinetic effects) by itself do not have a universal capacity to damp the development of thermal instabilities. This conclusion applies to practically important ranges of controlling parameters identified in^1^. Practical implication of that conclusion is that if temperature oscillations are observed in the storage of reactive material, an active intervention measures may need be taken to prevent autoignition and further full scale fire development.

Critical boundaries for thermal explosion have been quantified for different kinetic mechanisms. Scenarios of transition to instability have been qualitatively identified, and quantified in a number of specific examples. Instability develops through a number of oscillations in the vicinity of the critical boundary, and in monotonically growing fashion further away from the boundary. This is an entirely new finding never reported before for the problem under consideration.

The developed model has a wide range of applicability, e.g. to any quantity of the solid reactive materials (including large capacity storages that are of most practical interest), to a wide range of heat exchange regimes dictating the state of the system (from adiabatic to isothermal), to inverse problems (i.e. calculation of the heat removal rate required to prevent explosion).

Another advantage of the model is that it can easily be generalised, for example to any real profile of ambient temperature variations which deviates from ideal harmonic oscillations.

## Conclusions

The present paper considers the effects of reactant consumption on thermal explosion development in oscillating ambient conditions. Such effects are very important for evaluation of fire autoignition risks in storages of various solid materials subjected to daily and/or seasonal temperature variations.

Three different kinetic reactant consumption mechanisms are considered, specifically through the first order, second order and autocatalytic reactions. Thermal explosion conditions in all the cases are compared to the reference case of zero order kinetics where reactants consumption is neglected. Analysis of the latter case was reported by in the previous publication^1^ by the present author.

It is found that with any of the considered kinetic mechanism involved oscillations of ambient temperature may transform system from thermal equilibrium conditions to thermal explosion. Therefore, in general reactants consumption do not mitigate fire risks.

Critical boundaries for thermal explosion in the presence of kinetic effects are quantified. Critical conditions are described in terms of dependence of the critical amplitude of oscillations on the oscillation frequency. It is found that critical amplitude grows as a function of frequency in essentially linear manner.

In addition, effects of the two key non-dimensional parameters, namely the Todes number and Zeldovich number are investigated.

The principal and novel finding of the present paper is that oscillatory development of thermal explosion (i.e. infinite temperature growth through a number of oscillations during induction period, discovered in the previous publication^1^) does not represent exceptional, isolated case but rather is an inevitable mode of instability development upon crossing instability boundary in the phase space of controlling parameters. It is demonstrated that with any type of kinetic mechanisms involved both oscillatory and monotonic thermal instability development occur.

Practical implication of the present paper findings are in preventing fire and autoignition development risks in storages of various chemically active solid, granular or dispersed materials. Such storages are often subjected to the influence of daily and/or seasonal temperature variations. Calculations presented in the present paper cover practically important ranges of controlling parameters. As such, transition from stable to unstable thermal behaviour of the system upon development of ambient condition oscillations at its boundaries may well occur in practical scenarios and lead to solid fuel autoignition and development of accidental fires.

As an immediate practical implication, development of observable temperature oscillations in the stored materials indicate that active fire prevention measures may need be taken.

### Data availability

All data generated or analyzed during this study are included in this published article.
